# Breast Cancer with Subacute Progressive Neurological Symptoms due to Anti-Yo Antibody–Associated Paraneoplastic Neurological Syndrome: A Case Report

**DOI:** 10.70352/scrj.cr.26-0487

**Published:** 2026-09-02

**Authors:** Moe Tasaki, Misato Okazaki, Yoshiya Horimoto, Minori Kawabata, Mariko Yoshino, Chisaki Hao, Kotaro Iijima, Yuji Tomizawa, Taku Hatano, Goro Kutomi

**Affiliations:** 1 Department of Breast Oncology, Juntendo University Faculty of Medicine, Tokyo, Japan; 2 Department of Human Pathology, Juntendo University Faculty of Medicine, Tokyo, Japan; 3 Department of Breast Surgery and Oncology, Tokyo Medical University, Tokyo, Japan; 4 Department of Neurology, Juntendo University Faculty of Medicine, Tokyo, Japan

**Keywords:** paraneoplastic neurological syndrome, anti-Yo antibody, breast cancer, subacute neurological symptoms, tumor-directed treatment

## Abstract

**INTRODUCTION:**

Anti-Yo antibody–associated paraneoplastic neurological syndrome (PNS) is a rare immune-mediated disorder that often presents with subacute cerebellar symptoms and is frequently associated with breast and gynecological malignancies.

**CASE PRESENTATION:**

A woman in her 50s developed subacute progressive neurological symptoms, including dizziness, diplopia, dysphagia, gait disturbance, ataxia, and nystagmus. Brain MRI showed no obvious abnormalities. Although Miller–Fisher syndrome was initially suspected, intravenous immunoglobulin therapy had limited efficacy, and serum anti-Yo antibodies were subsequently detected. Further investigation revealed human epidermal growth factor receptor 2–positive breast cancer with axillary lymph node metastasis. Because her neurological symptoms rapidly progressed and her performance status deteriorated, neoadjuvant chemotherapy was considered difficult. Left mastectomy with axillary lymph node dissection was therefore performed as prompt tumor-directed treatment. The final pathological stage was pT1aN1aM0, stage IIA. Postoperatively, additional immunomodulatory treatments and rehabilitation were provided for the neurological deficits.

**CONCLUSIONS:**

This case highlights the importance of considering anti-Yo antibody–associated PNS in patients with unexplained subacute progressive neurological symptoms, even when brain imaging shows no clear abnormalities. Early recognition of PNS and prompt screening for an underlying malignancy, including breast cancer, are essential to allow timely tumor-directed treatment before severe neurological deficits become established.

## Abbreviations

CA15-3
: cancer antigen 15-3
CDR2L
: cerebellar degeneration-related protein 2-like
CEA
: carcinoembryonic antigen
ER
: estrogen receptor
HER2
: human epidermal growth factor receptor 2
IgG
: immunoglobulin G
IMP
: N-isopropyl-p-[^123^I]iodoamphetamine
PCD
: paraneoplastic cerebellar degeneration
PgR
: progesterone receptor
PNS
: paraneoplastic neurological syndrome

## INTRODUCTION

PNS is a group of disorders that causes various neurological manifestations in patients with cancer through immune responses directed against the tumor.^1,2)^ Autoimmune responses against antigens shared by tumor cells and neural tissues are considered central to its pathogenesis, and the involvement of anti-neuronal antibodies is well recognized. PNS includes several clinical phenotypes, each of which is often associated with characteristic autoantibodies and relatively specific underlying tumors.^2)^ In many cases, neurological symptoms precede the diagnosis of the primary tumor and present as subacute progressive neurological impairment.^3,4)^ Here, we report a case of breast cancer with subacute progressive neurological symptoms due to anti-Yo antibody–associated PNS.

## CASE PRESENTATION

A woman in her 50s developed an unusual sensation in her head and pain in the left upper arm in late October and visited a local neurosurgery clinic. However, brain MRI showed no obvious abnormalities. In early November, she was referred to the orthopedic department of Juntendo University Hospital with a chief complaint of left shoulder pain, but blood tests, including markers related to collagen vascular diseases, showed no apparent abnormalities. MRI of the left shoulder joint also revealed no abnormalities. Cervical spine MRI showed mild cervical spondylosis at the C5/6 level, but no clear nerve root compression was identified. She was also evaluated by the cardiology department, where neither electrocardiographic abnormalities nor arteriosclerotic changes were observed, and a cardiogenic cause was considered unlikely. She was followed up with symptomatic treatment, but her symptoms tended to worsen. She subsequently visited the ophthalmology, otorhinolaryngology, and general medicine departments on her own initiative and underwent further examinations, but no obvious abnormalities were found in any of these evaluations. In December, she developed dizziness, diplopia, and a sensation of difficulty swallowing, followed by gait disturbance, and was referred to the neurology department of our hospital. Her medical history included resection of a right parotid gland tumor and catheter ablation for premature ventricular contractions. Her family history included parkinsonism in her mother and lung cancer in her father.

**Fig. 1:**
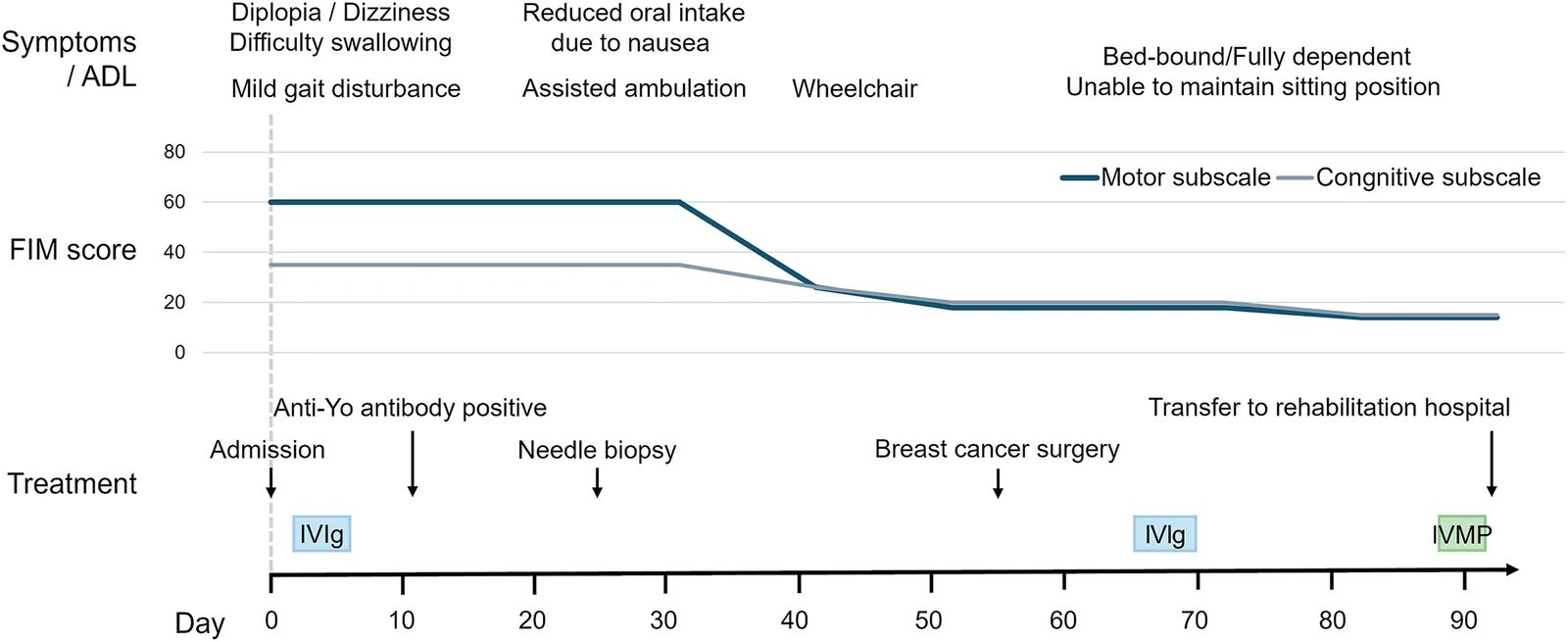
Summary of the clinical course. Clinical course after admission to the Department of Neurology, showing changes in neurological symptoms, ADL, FIM scores, and the timing of therapeutic interventions. Day 0 indicates the date of admission to the Department of Neurology. FIM is a validated scale assessing the level of independence in ADL (range, 18–126; higher scores indicate greater independence). FIM, Functional Independence Measure; IVIg, intravenous immunoglobulin; IVMP, intravenous methylprednisolone

Neurological examination revealed decreased tendon reflexes in both the upper and lower limbs, predominantly distal sensory impairment, ataxia, diplopia, gaze-evoked nystagmus, and decreased vibratory sensation. Blood tests showed no obvious findings suggestive of endocrine abnormalities, vitamin deficiency, infection, autoimmune disease, or malignancy. Cerebrospinal fluid examination revealed mild increases in protein level (117 mg/dL; reference range, 15–45 mg/dL) and cell count (18/μL; reference range, 0–5/μL). Contrast-enhanced brain MRI showed no obvious abnormal signal intensity. IMP single-photon emission CT showed a relative increase in cerebellar blood flow. Although nerve conduction studies showed no abnormalities, the clinical course and neurological findings led to an initial suspicion of Miller–Fisher syndrome. Intravenous immunoglobulin therapy was administered; however, the clinical response was limited, and serum anti-GQ1b antibodies were not detected. The subsequent clinical course and changes in functional status, assessed using the Functional Independence Measure (FIM),^5)^ are shown in **Fig. 1**. On the other hand, because subacute progressive cerebellar symptoms, including ataxia and nystagmus, had been present from the initial presentation, PNS was also considered in the differential diagnosis. Subsequent serum antibody testing revealed positivity for anti-Yo antibodies. Given the limited response to intravenous immunoglobulin therapy and the presence of anti-Yo antibodies, further investigation for breast cancer and gynecological malignancies, which are strongly associated with these antibodies, was planned with PNS in mind.

**Fig. 2:**
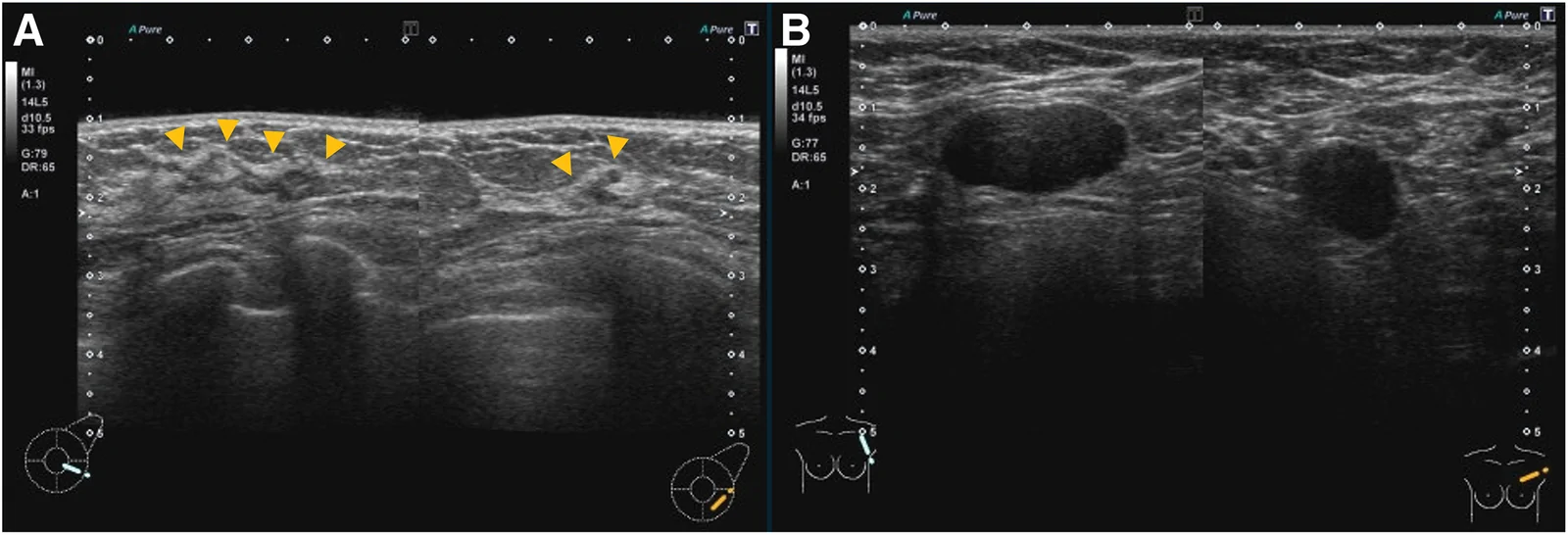
US findings. (**A**) Breast ultrasonography showed an indistinct hypoechoic lesion measuring 20 × 9 mm in the lower outer quadrant of the left breast (orange arrowheads). (**B**) Enlarged lymph nodes were detected in the axilla.

**Fig. 3:**
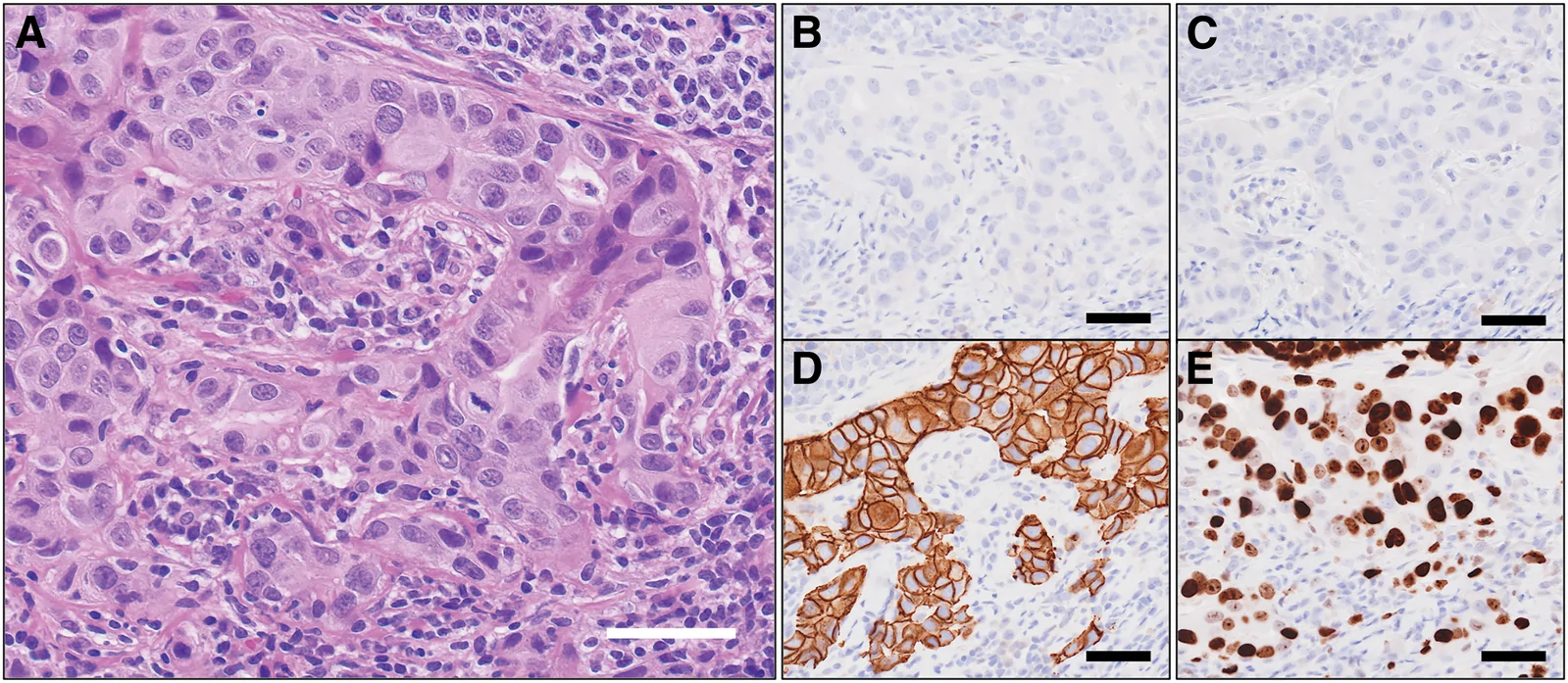
Histological findings of the biopsy specimen of the breast tumor. Histological features of the tumor on hematoxylin and eosin staining are shown in (**A**). Immunohistochemical results for ER (**B**), PgR (**C**), HER2 (**D**), and Ki67 (**E**) are also shown. All scale bars indicate 50 μm. ER, estrogen receptor; HER2, human epidermal growth factor receptor 2; PgR, progesterone receptor

Whole-body CT revealed left axillary lymph node enlargement, but no other obvious neoplastic lesions, including in the breasts, were identified. In late December, the patient was referred to Department of Breast Oncology for further breast evaluation. On physical examination, no obvious lesion was palpable. Mammography showed no abnormal findings in either breast. The breast ultrasonographic findings are shown in **Fig. 2**. In the lower outer quadrant of the left breast, an indistinct hypoechoic area measuring 20 × 9 mm with increased vascularity and posterior acoustic attenuation was observed; the findings suggested mastopathy or ductal carcinoma *in situ*. Multiple enlarged lymph nodes, up to 23 mm in maximum diameter, were also observed at levels I–II of the left axilla. Blood tests, including complete blood count and biochemical tests, showed no obvious abnormalities, and the tumor markers CEA and CA15-3 were both within the reference ranges. Core needle biopsy of the left breast lesion revealed invasive ductal carcinoma: nuclear grade 2, histological grade II, ER-negative, PgR-negative, HER2-positive, and Ki67 labeling index of 60% (**Fig. 3**). Biopsy of the enlarged left axillary lymph node confirmed metastasis from breast cancer.

**Fig. 4:**
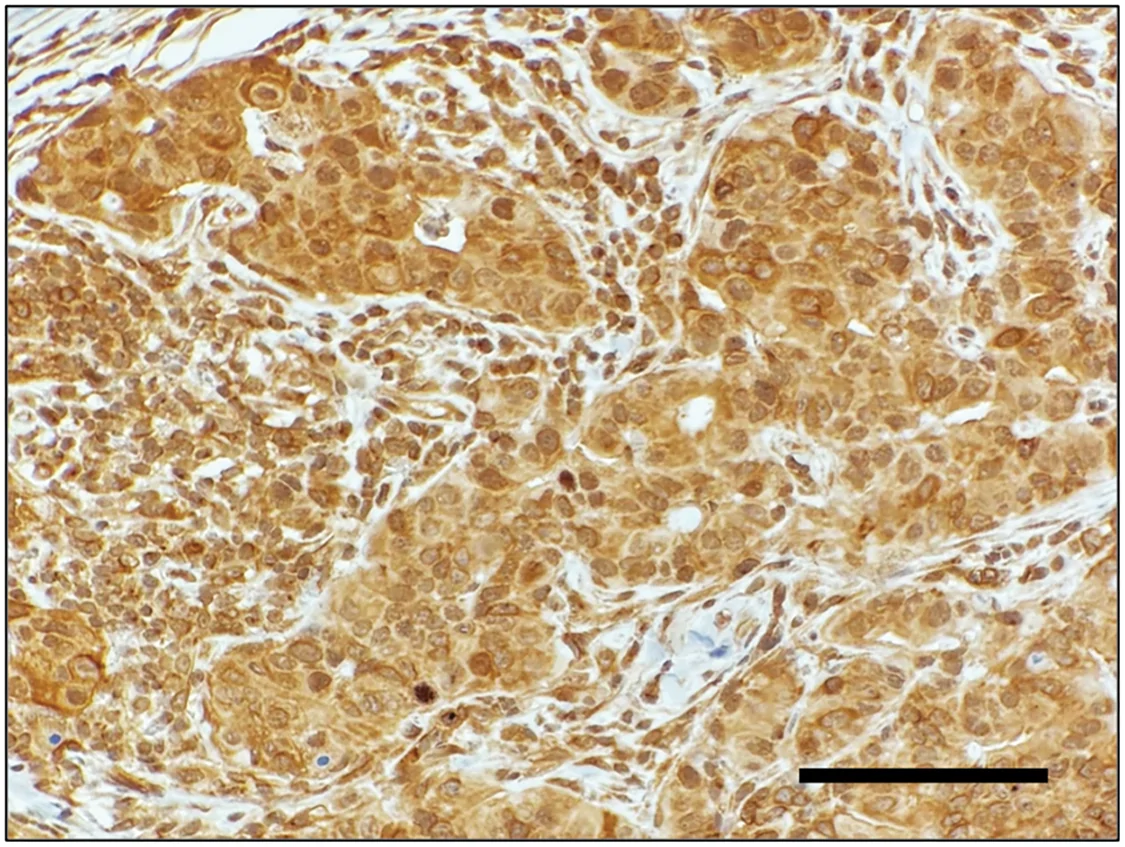
Immunohistochemical staining for CDR2L in the breast tumor. Immunohistochemical staining for CDR2L, the major target antigen of anti-Yo antibodies, was performed using a monoclonal anti-CDR2L antibody (clone 2E12D10; Proteintech, Rosemont, IL, USA). The tumor cells showed cytoplasmic staining for CDR2L. A scale bar indicates 100 μm. CDR2L, cerebellar degeneration-related protein 2-like

During the diagnostic work-up, her neurological symptoms progressively worsened, with marked deterioration in physical function and difficulty in communication. Although neoadjuvant chemotherapy was considered as one of the treatment options, her performance status was deteriorating on a daily basis, making its administration difficult. Therefore, left mastectomy with axillary lymph node dissection was performed in the following February with the aim of suppressing further neurological progression. In the surgical specimen, metastasis was identified in one of the 13 dissected lymph nodes. The primary lesion consisted predominantly of an intraductal component extending over an area of 13 × 11 mm, and only microinvasion measuring 0.2 mm in diameter was observed. However, because the biopsy specimen had shown an invasive carcinoma component measuring up to 3 mm, the final pathological stage was diagnosed as pT1aN1aM0, stage IIA. As an exploratory analysis, immunohistochemistry for CDR2L, which is considered the major target antigen of anti-Yo antibodies,^6)^ was performed on the biopsy specimen, and the tumor cells showed diffuse and homogeneous cytoplasmic staining throughout the tumor, without apparent regional variation (**Fig. 4**).

The postoperative course was uneventful; however, no clear improvement in the neurological symptoms was observed, as expected, and she continued to have impaired physical function and difficulty in communication. Adjuvant chemotherapy for breast cancer was not administered in consideration of her general condition. Additional pharmacological treatments, including intravenous immunoglobulin therapy and steroid pulse therapy, were administered with the aim of improving the neurological symptoms, but no improvement was achieved. Therefore, priority was placed on maintaining her remaining functional status, and she was transferred to another hospital for rehabilitation.

## DISCUSSION

PNS is classified into several representative clinical phenotypes based on their neurological manifestations, and each phenotype is known to be associated with characteristic autoantibodies and underlying malignancies.^2)^ One such phenotype is PCD.^2,4)^ Anti-Yo antibody–associated PNS commonly presents clinically as PCD, which is characterized by subacute cerebellar ataxia progressing over several weeks to months. In addition, anti-Yo antibody–associated PNS is commonly associated with breast and gynecological malignancies.^4,7)^

Anti-Yo antibody–associated PNS often lacks clear abnormalities on imaging studies such as MRI, making diagnosis based on imaging findings alone difficult.^8)^ In addition, onconeural antibodies are not detected in all patients, and PNS cannot be excluded solely on the basis of negative antibody testing.^9–11)^ Therefore, when unexplained subacute progressive neurological impairment is observed, it is important not to rely solely on imaging findings or antibody testing, but to consider the possible presence of an underlying malignancy and to include PNS in the differential diagnosis from an early stage.^2)^ Neurological deficits that have already developed as a result of Anti-Yo antibody–associated PNS are generally considered difficult to reverse,^4,8,12)^ and previous reports have also described clinical courses in which no clear neurological improvement was achieved even after therapeutic intervention.^13)^ Previous studies have suggested that the poor reversibility of anti-Yo antibody–associated PCD may be explained, at least in part, by CD8-positive T cell–mediated cytotoxicity, which induces irreversible injury and loss of cerebellar Purkinje cells.^4,8)^ Because Purkinje cells have limited regenerative capacity, neurological deficits may persist once neuronal damage has occurred, underscoring the importance of early diagnosis and prompt treatment.^14)^ Hence, tumor control may contribute to stabilization or suppression of neurological progression, and even in anti-Yo antibody–associated PNS, in which severe functional impairment often remains, stabilization or suppression of neurological symptoms may be achieved in some cases.^4,12)^

Several case reports have described breast cancer associated with anti-Yo antibody–positive PNS.^8,13,15)^ Rojas-Marcos et al. analyzed 27 patients with breast cancer associated with anti-Yo-positive PNS presenting as PCD and reported that HER2 overexpression was observed at a high frequency of 96%.^15)^ Our case was also HER2-positive. Furthermore, Peter et al. analyzed breast cancers associated with anti-Yo-related PCD and reported that these tumors exhibited mutation, amplification, and overexpression of CDR2L, which is considered the major target antigen of anti-Yo antibodies.^6)^ They also observed a strong intratumoral immune reaction predominantly composed of IgG-positive plasma cells. These findings suggest that CDR2L alterations and intratumoral humoral immune responses in HER2-positive breast cancer may be involved in the development of anti-Yo antibody–associated PNS. In our case, exploratory immunohistochemical analysis also demonstrated cytoplasmic CDR2L expression in the tumor cells. However, because no comparison with other breast cancers was performed and CDR2L can also be expressed in non-neoplastic cells, the significance of this finding remains unclear and warrants further investigation.

## CONCLUSIONS

In the present case, the interval from diagnosis to surgery was relatively short, and there was no apparent delay in treatment. Therefore, the lack of neurological improvement in this case was considered to be largely attributable to the nature of this disease, which often causes irreversible neurological damage. Anti-Yo antibody–associated PNS is a rare condition, and neurological symptoms often precede the diagnosis of malignancy. Therefore, when unexplained subacute progressive neurological impairment is observed, it is important to consider anti-Yo antibody–associated PNS, particularly when peripheral neuropathy and cerebellar ataxia are present. Autoantibody testing and screening for underlying malignancies, including breast cancer, should then be performed promptly.
